# Nanoformulation of Talazoparib Increases Maximum Tolerated Doses in Combination With Temozolomide for Treatment of Ewing Sarcoma

**DOI:** 10.3389/fonc.2019.01416

**Published:** 2019-12-17

**Authors:** Paige Baldwin, Rostislav Likhotvorik, Nabeela Baig, Jodie Cropper, Ruth Carlson, Raushan Kurmasheva, Srinivas Sridhar

**Affiliations:** ^1^Department of Bioengineering, Northeastern University, Boston, MA, United States; ^2^Greehey Children's Cancer Research Institute, San Antonio, TX, United States; ^3^Department of Molecular Medicine, The University of Texas Health Science Center at San Antonio, San Antonio, TX, United States; ^4^Department of Physics, Northeastern University, Boston, MA, United States; ^5^Division of Radiation Oncology, Harvard Medical School, Boston, MA, United States

**Keywords:** talazoparib, temozolomide, nanoparticle, combination therapy, Ewing sarcoma

## Abstract

The Pediatric Preclinical Testing Program previously identified the PARP inhibitor talazoparib (TLZ) as a means to potentiate temozolomide (TMZ) activity for the treatment of Ewing sarcoma. However, the combination of TLZ and TMZ has been toxic in both preclinical and clinical testing, necessitating TMZ dose reduction to ~15% of the single agent maximum tolerated dose. We have synthesized a nanoparticle formulation of talazoparib (NanoTLZ) to be administered intravenously in an effort to modulate the toxicity profile of this combination treatment. Results in Ewing sarcoma xenograft models are presented to demonstrate the utility of this delivery method both alone and in combination with TMZ. NanoTLZ reduced gross toxicity and had a higher maximum tolerated dose than oral TLZ. The dose of TMZ did not have to be reduced when combined with NanoTLZ as was required when combined with oral TLZ. This indicated the NanoTLZ delivery system may be advantageous in decreasing the systemic toxicity associated with the combination of oral TLZ and TMZ.

## Introduction

Ewing sarcoma (ES) comprises the fourth most common highly malignant childhood solid tumor (1, 2). Most patients are diagnosed between 10 and 20 years old and 70% of patients will be cured with intensive chemotherapy regimens (3). However, 25% of patients present with metastatic disease at the time of diagnosis and the prognosis for these cases is unfavorable with 5-year survival rates around 30% (1). Advances in chemotherapy regimens, radiotherapy, and surgery have shown dramatic improvements in the management of local tumors. These advances have come in the form of dose intensification and compression, with few advances in identifying new compounds for treating these tumors. However, very little progress has been made in the treatment of advanced or metastatic disease.

ES is defined by a tumor-specific chromosomal translocation (4–6). In approximately 85% of all tumors, the EWSR1 gene on chromosome 22 is fused to FLI1, a member of E26 transformation-specific sequence (ETS) family of transcription factors, on chromosome 11. In the remaining 15% of ES tumors, the EWSR1 is fused to other members of the ETS family, mostly the ERG gene on chromosome 21 (7, 8). It has been shown recently, that ETS transcription factors interact with Poly-ADP ribose polymerase 1 (PARP1), the founding member of the DNA damage repair superfamily of enzymes (9, 10). It is postulated that PARP1 is a direct transcriptional target of EWSR1-FLI1, and it interacts with EWSR1-FLI1 or EWSR1-ERG fusion proteins in a feed-forward loop to enhance oncogenic transcription factor function (9). Further, ETS gene fusions induce DNA double-strand breaks (9, 11). Thus, it is postulated that inhibiting PARP activity has a selective effect on ES cells through downregulating the activity of the oncogenic EWSR1-FLI1 fusion protein, leading to selective hypersensitivity of ES cell lines to PARP inhibitors as was identified using a genomic screen (12). However, despite the promising activity of PARP inhibitors as single agents *in vitro*, they have shown only modest activity in *in vivo* models without defects in homologous recombination (10).

Talazoparib (TLZ), a potent PARP inhibitor, was evaluated as a single agent in 44 xenograft models representing childhood solid tumors, but only two models demonstrated regression (10). There was no activity in ES xenografts, which appears to be reflective of clinical activity, since a phase II clinical trial of the PARP inhibitor olaparib showed no activity in ES tumors (13). Preclinical studies indicate the combination of PARP inhibitors with chemotherapy agents that damage DNA induces synergy *in vitro* and promising activity in xenograft models (9, 10, 14–16). It has been shown *in vitro* that the potency of temozolomide (TMZ) can be potentiated up to 40-fold through inhibition of PARP by TLZ, not only in ES cells (17). In our previous study, neither TLZ nor TMZ as single agents yielded biologically significant anti-tumor activity against ES xenografts, while the combination of the two agents led to dramatic regression in 5 of the 10 ES xenograft models (17). However, this combination was toxic, necessitating a reduction of TMZ to ~15% of its single agent maximum tolerated dose (MTD). Results of a recent phase I/II clinical trial to assess the combination of TMZ and TLZ in pediatric patients with recurrent disease (NCT02116777) suggests a similar TMZ dose reduction is required to make this combination tolerable.

Nanoparticles have been widely studied as drug delivery systems due to their inherent ability to reduce toxicity while maintaining therapeutic efficacy (18, 19). Nanoparticles can be administered intravenously meaning the drug is 100% available in the vasculature. In contrast, oral drugs must cross the gastro-intestinal barrier, a rate limiting step for drug absorption, and subsequently undergo first-pass metabolism. Tumors are known to rapidly induce blood vessel growth to supply them with nutrients, resulting in a highly disorganized vascular network with compromised lymphatic draining. This leaky vasculature, and poor lymphatic drainage, aids in the enhanced permeability and retention (EPR) effect, whereby nanoparticles are more likely to extravasate and remain in tumor tissue instead of healthy tissues (20). A nanoformulation of TLZ (NanoTLZ) has been developed and shown to be more effective than oral TLZ at delaying ascites formation in a disseminated ovarian cancer model (21). Additionally, NanoTLZ induced greater regression than both oral and intravenous (IV) TLZ in a *BRCA1* deficient model of breast cancer without any signs of toxicity (22). Therefore, we sought to utilize NanoTLZ in combination with TMZ to more effectively treat ES. We hypothesized that NanoTLZ would be less toxic than oral TLZ, consequently allowing for combination with TMZ at doses closer to the single agent MTD. Lowering the toxicity of the combination is expected to provide more effective treatment for these tumors.

## Materials and Methods

### Synthesis and Characterization of NanoTLZ

Formulation and characterization of NanoTLZ have been previously reported (21, 22). Briefly, fixed ratios of 1, 2-dipalmitoyl-sn-glycero-3-phosphocholine (DPPC), 1,2-dioleoyl-3-tri methyl-ammonium-propane (chloride salt) (DOTAP), cholesterol, and 1,2-distearoyl-sn-glycero-3 phosphoethanolamine-N-[methoxy(polyethyleneglycol)-2000 (DSPE-PEG2000), and TLZ were mixed in chloroform and evaporated to form a thin film. The film was hydrated with phosphate buffered saline (PBS) at 50°C and sized using bath sonication for 20 min. Nanoparticles were dialyzed against PBS and additional non-encapsulated drug which is insoluble in aqueous media was removed via syringe filter (23). Vehicle nanoparticles were prepared following the same protocol without the addition of TLZ. Fluorescently labeled nanoparticles were prepared by including Cyanine 5 (Cy5) in the lipid mixture.

Each batch was characterized in regards to size and zeta potential using a Brookhaven 90Plus analyzer equipped with ZetaPALS. The concentration of encapsulated TLZ was measured by lysing nanoparticles with methanol for analysis via high performance liquid chromatography as previously described.

### *In vitro* Assessment of NanoTLZ

ES-6, ES-7, EW-8 ES cells have been previously determined to be sensitive to single agent TLZ and therefore, were utilized to ensure NanoTLZ was as effective as free TLZ *in vitro* (10). TC-71 cell line is not sensitive to single agent TLZ but has been previously shown that treatment with a low dose of TLZ can potentiate killing by TMZ, therefore, TC-71 was further used to assess the ability of NanoTLZ to potentiate the effect of TMZ by treating cells with the IC_10_ of either TLZ or NanoTLZ and assessing dose response to TMZ. The Alamar Blue® assay was used to assess cell viability (*BioRad*). Cells were seeded to reach 20–40% confluency. TLZ, NanoTLZ, or TMZ were added to wells 24 h after cell seeding, and incubated for 96 h. Following the 96 h incubation of cells in 24-well plates, 10% v/v of Alamar Blue was added and fluorescence was measured after 4 h (excitation 530 nm, emission 590 nm). Wells containing RPMI 1640 (*Hyclone*), 10%FBS (*Sigma*) and untreated cells, 10% v/v Alamar blue, were used as positive controls. Wells with culture medium without cells containing 10% v/v Alamar Blue were assays as negative controls. Fluorescence was recorded on the Spectra Max plate reader, with the Alamar Blue protocol provided by *Softmax Software*. All experiments were performed in triplicate. Statistical analysis and curve plotting (3-parameter polynomial analysis) were performed using standard equations included in the GraphPad Prism 7.0c package (*GraphPad Software Inc., USA*).

### Immunoblotting

Mice harboring KT-10 xenografts were treated with 0.165 mg/kg TLZ BID x5 PO or 0.33 mg/kg NanoTLZ SID, IV, on days 1, 3, and 5. Tumors were collected from 3 mice/group for immunoblotting. Cells were lysed using RIPA buffer (*89900, Pierce*) according to standard protocols. Samples were separated on a 4–12% gradient gel (*NP0321, Invitrogen*) and then transferred onto a PVDF or nitrocellulose membrane. Membranes were blocked with 3% BSA in TBS-T for 1 h at room temperature, then incubated with primary PARP1/cleaved PARP1 or GAPDH antibodies overnight (*Cell Signaling Technology*). After secondary antibody incubation and washing, membranes were developed using enhanced chemiluminescence (*NEL103001EA, PerkinElmer*).

### MTD of NanoTLZ

All animal studies and procedures, unless otherwise stated, were conducted in accordance with the Institutional Animal Care and Use Committee (IACUC) reviewed and approved at the University of Texas Health San Antonio.

For evaluation of toxicity, non-tumored C.B.17SC *scid-/-* female mice (*Taconic Farms, NY*) were administered 0.125, 0.25, 0.5, or 1 mg/kg NanoTLZ IV either on days 1, 3, and 5 or daily for 5 days to assess the single agent MTD (*n* = 3/group). To assess the combination MTD, mice were treated with 0.5 mg/kg NanoTLZ IV daily for 5 days combined with 5, 10, 20, 30, or 40 mg/kg TMZ oral gavage daily for 5 days. A second combination assessed 1.0 mg/kg NanoTLZ IV on days 1, 3, and 5 combined with 50 mg/kg TMZ oral gavage for 5 days. Body weight was measured daily for 21 days. Loss of more than 20% of the initial body weight was considered toxic and the next lower dose would be considered the MTD.

### *In vivo* Localization

This animal study was performed in accordance with protocols approved by the IACUC at Northeastern University. NCr-nu/nu mice were implanted with 10^6^ MDA-MB-231-D3H2LN cells in matrigel. When tumors reached ~100 mm^3^ a single dose of NanoTLZ-Cy5 IV was administered (*n* = 3). Twenty-four hours after administration fluorescent imaging was completed using an IVIS Lumina II. The primary image was collected at an excitation wavelength of 640 nm, the background image was excited at 570 nm and the collected emission was 695–770 nm.

### Efficacy of NanoTLZ Monotherapy

The KT-10 Wilms tumor PDX model was used to assess the activity of NanoTLZ. This model has a PALB2 mutation, hence is defective in homologous recombination and is sensitive to TLZ (10). The PPTP previously identified relevant doses of free TLZ for this model which were used in this study (10). MTD testing in non-tumored mice as mentioned in section MTD of NanoTLZ was used to identify the NanoTLZ dose. C.B.17SC *scid-/-* mice implanted with KT-10 xenografts were treated with 1 mg/kg NanoTLZ or vehicle (empty nanoparticles) IV on days 1, 3, and 5; or with 0.1625 or 0.33 mg/kg free TLZ by oral gavage daily for 5 days (*n* = 8–10/group). Tumor diameters were measured weekly using digital calipers, and body weights were measured. Animals were euthanized when tumor volume reached 400% of the volume at start of treatment. Tumor responses were classified into 5 categories: progressive disease (PD), >25% increase in tumor volume; stable disease (SD), <25% increase in tumor volume and <50% regression; partial response (PR), regression ≥50% for at least one time point; complete response (CR), no measurable tumor (<0.04 cm^3^); and maintained complete response (MCR), tumor volume <0.1 cm^3^ at the end of the study (17).

### NanoTLZ in Combination With TMZ

C.B.17SC *scid-/-* mice implanted with TC-71 ES xenografts were utilized to assess efficacy of NanoTLZ in combination with TMZ. The TC-71 model was selected as it does not respond to either TLZ or TMZ as a single agent but is responsive to the combination (17). Mice were treated with 1 mg/kg NanoTLZ IV on days 1, 3, and 5 combined with TMZ 50 mg/kg oral gavage (PO) daily for 5 days (*n* = 10/group). Tumor dimensions and body weight were measured twice weekly. Animals were euthanized when tumor volume reached 400% of the volume at start of treatment. Tumor responses were as described above.

### Statistical Analysis

All *in vitro* data were plotted as mean ± SD. The statistical significance of *in vitro* data was determined by using Student's *t*-tests with α = 0.05 for significance. *In vivo* efficacy data were plotted individually or as median relative tumor volume. Toxicity data were plotted as mean ± SEM. The log-rank test with the Bonferroni correction for multiple comparisons was used to assess family-wise significance of survival curves. All statistical testing computed with Prism 7.

## Results

### Validation of NanoTLZ

**Figure 1:**
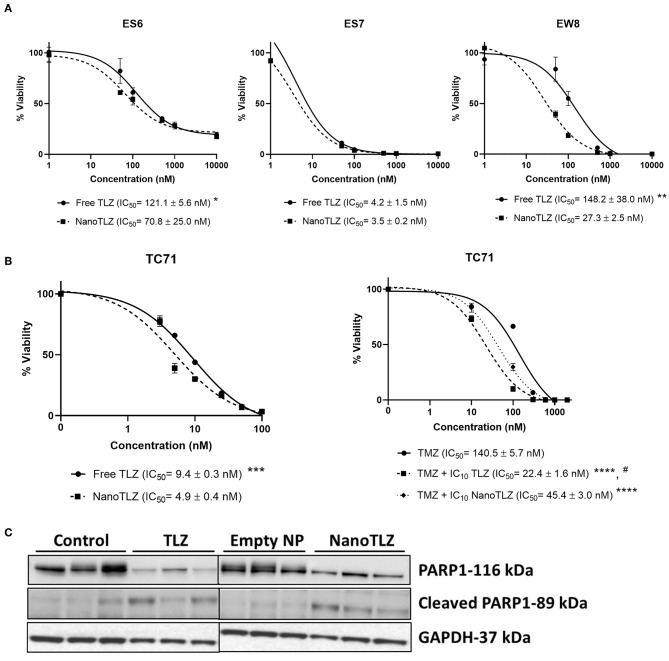
NanoTLZ is as efficacious at free TLZ *in vitro*. ES-6, ES-7, and EW-8 cell lines were treated with TLZ or NanoTLZ for 72 h and viability was assessed by Alamar Blue (*n* = 3/line). IC_50_ plots for TLZ and NanoTLZ in ES-6, ES-7, and EW-8 cell lines **(A)**. Statistical significance of TLZ and NanoTLZ IC_50_ values were assessed for each cell line by Student's *t*-tests with α = 0.05 for significance; *p < 0.05 vs. NanoTLZ; **p < 0.01 vs. NanoTLZ.TC-71 cells were treated with the IC_10_ of TLZ or NanoTLZ and dose response to TMZ was measured using Alamar Blue. Potentiation of TMZ activity in TC-71 cells combined with TLZ or NanoTLZ **(B)**. Statistical significance of TMZ, TLZ, and NanoTLZ combination IC_50_ values were assessed by one way ANOVA followed by Tukey's test for multiple comparisons; ***p < 0.001 vs. NanoTLZ; ****p < 0.0001 vs. TMZ; ^#^*p* < 0.001 vs. TMZ + NanoTLZ. Mice harboring KT-10 xenografts were treated with 0.165 mg/kg TLZ BID x5 PO or 0.33 mg/kg NanoTLZ SID, IV, on days 1, 3, and 5. NanoTLZ and TLZ demonstrate inhibitory effect on total PARP1 protein levels in KT-10 tumor xenograft (*n* = 3/group) **(C)**.

NanoTLZ has been previously optimized and found to have stable physicochemical properties which impart advantages for nanoparticle mediated delivery of TLZ (21, 22). In order to validate NanoTLZ efficacy *in vitro*, ES-6, ES-7, and EW-8 ES cells were treated with either TLZ or NanoTLZ. All cell lines were found to have lower IC_50_ values in response to NanoTLZ (Figure 1A). Both NanoTLZ and TLZ were also found to potentiate the effect of TMZ in TC-71 cells (Figure 1B).

The PARP1 total and cleaved protein levels were evaluated in KT-10 xenograft model. This Wilms tumor model has shown previously to be sensitive to the free TLZ treatment, hence, it was used here to determine the effect of NanoTLZ on the target protein (10). As shown on Figure 1C, PARP1 levels were significantly reduced in tumor cells treated with TLZ or NanoTLZ compared to control or empty nanoparticle. The levels of cleaved PARP1 remained low overall in all treatment groups with slight increases of cleaved PARP1 in TLZ and NanoTLZ treated cells indicating that neither of the drugs had strong apoptotic effect at the clinically relevant doses used before (0.165 mg/kg TLZ BID x5 PO and 0.33 mg/kg NanoTLZ SID, days 1, 3, and 5, IV) (17).

**Figure 2:**
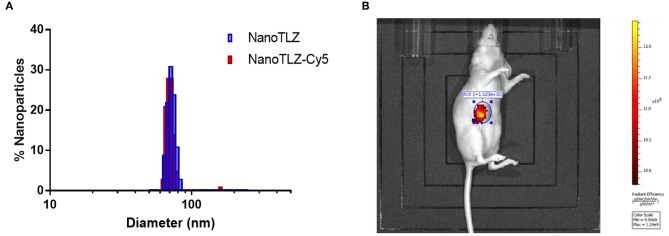
NanoTLZ preferentially accumulates within tumors. The size distribution of NanoTLZ as measured by DLS does not change with the addition of Cy5 dye **(A)**. Live animal fluorescent imaging demonstrated NanoTLZ-Cy5 accumulates in the tumor 24 h after injection (*n* = 3) **(B)**.

The efficacy of NanoTLZ relies on the EPR effect; therefore, it was crucial to ensure the particles accumulate at the tumor. NanoTLZ was fluorescently labeled *via* the encapsulation of Cyanine 5 (Cy5) dye. The addition of Cy5 did not significantly alter the diameter, polydispersity, or zeta potential of NanoTLZ, and therefore was optimal to assess tumor accumulation (Figure 2A). Twenty-four hours after a single dose of NanoTLZ-Cy5 was administered, fluorescence was observed localized to the tumor via live animal imaging (Figure 2B).

### NanoTLZ Monotherapy

Toxicity testing was conducted to assess the MTD of single agent NanoTLZ. Doses of up to 1 mg/kg NanoTLZ (IV) administered daily (SID) on days 1, 3, and 5 and for 5 consecutive days were tolerated with no appreciable weight loss (data not shown). Therefore, 1 mg/kg on days 1, 3, and 5 was chosen to compare to oral TLZ therapy since the nanoformulation was expected to have a longer circulation time, and not require daily dosing.

**Figure 3:**
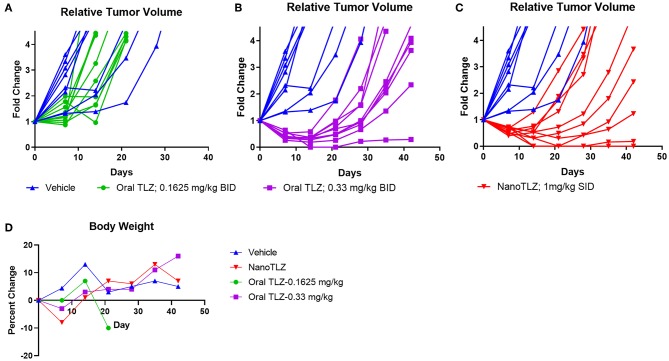
KT-10 xenografts exhibit a dose dependent response to TLZ. Animals bearing KT-10 xenografts were treated with either 0.1625 mg/kg **(A)**, 0.33 mg/kg oral TLZ BID x 5 **(B)**, or 1.0 mg/kg NanoTLZ (IV) SID x 5 **(C)** and relative tumor volume was plotted for each animal (*n* = 8–10/group). Percent change in weight during and after treatment **(D)**.

As mentioned earlier, the KT-10 Wilms tumor PDX model has a PALB2 mutation, hence is defective in homologous recombination and is sensitive to TLZ (10). Animals bearing KT-10 xenografts were treated with either 0.1625 mg/kg or 0.33 mg/kg free TLZ (PO) administered twice daily (BID) for 5 days. These doses were selected based on our previous testing of free TLZ (10). KT-10 tumors responded to TLZ treatment in a dose-dependent manner (Figures 3A,B). All tumors responded to oral TLZ and NanoTLZ therapy (Figures 3B,C). Most tumors in both treatment groups exhibited a partial response (PR) to therapy. However, 2/10 (20%) of tumors treated with NanoTLZ exhibited a complete response (CR), and 1/10 (10%) maintained complete response (MCR) over the course of the study. In contrast, only 10% of tumors treated with oral TLZ exhibited a CR to treatment. None of the treatments elicited significant weight loss throughout the course of the study (Figure 3D).

### NanoTLZ Combined With TMZ

**Figure 4:**
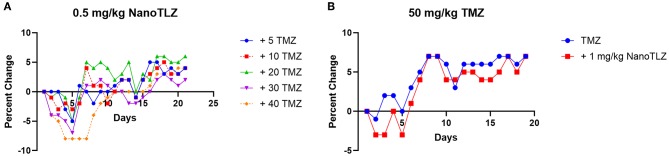
NanoTLZ and TMZ are tolerable at higher doses than previously established with TLZ and TMZ. Tumor free mice (*n* = 3/group) were treated with 0.5 mg/kg NanoTLZ SID x5 in combination with increasing doses of TMZ and body weight was measured daily for 21 days **(A)**. An alternative schedule assessed weight change during treatment with 50 mg/kg TMZ for 5 days and 1.0 mg/kg NanoTLZ on days 1, 3, and 5 **(B)**.

**Figure 5:**
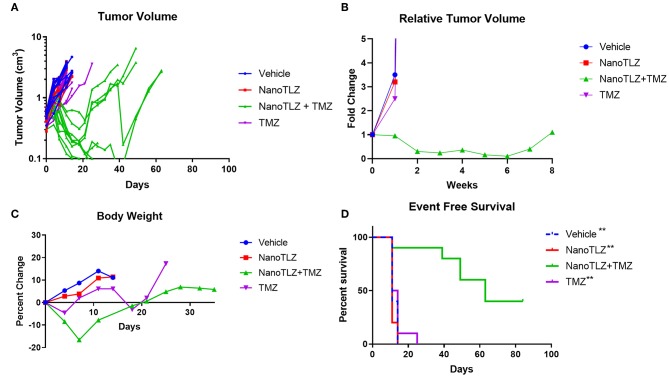
TC-71 xenografts are responsive to the combination of NanoTLZ + TMZ. Mice bearing TC-71 xenografts (*n* = 10/group) were treated with 1 mg/kg vehicle or NanoTLZ (IV) on days 1, 3, and 5, 50 mg/kg TMZ (PO) on days 1–5, or the combination of the two and tumor volume was monitored twice weekly **(A)**. Median relative tumor volume during 8 weeks of treatment **(B)**. Change in body weight during and up to 30 days after treatment **(C)**. Kaplan-Meier survival of TC-71 xenografts for 12 weeks after treatment initiation **(D)**. Statistical significance assessed via the log-rank test followed by the Bonferroni correction for multiple comparisons, **p < 0.01 vs. NanoTLZ+TMZ.

We previously established TC-71 xenografts are sensitive to the combination of TLZ and TMZ and therefore sought to explore the effect of utilizing NanoTLZ in combination with TMZ. Toxicity testing demonstrated the combination of NanoTLZ and TMZ daily for 5 days resulted in an average loss of ~8% body weight at the highest dose of each drug (Figure 4A). The combination of 1 mg/kg NanoTLZ on days 1, 3, and 5 and 50 mg/kg TMZ daily for 5 days resulted in an average loss of 3% body weight, therefore, this regimen was chosen for efficacy testing (Figure 4B). Treatment with 1 mg/kg NanoTLZ or empty nanoparticles IV on days 1, 3, and 5 did not yield any antitumor response (Figures 5A,B). Single agent TMZ, 50 mg/kg PO daily for 5 days, also was not active in this model as evidenced by the median fold change in tumor volume (Figure 5B). The combination of NanoTLZ+TMZ was active with all tumors initially responding to the treatment (Figure 5A). Progressive disease (PR) was observed in both single agent control arms, while 4/10 (40%) of tumors exhibited a PR to the combination and an additional 40% of tumors maintained a CR.

The combination therapy did elicit acute weight loss of 16.6% during the treatment cycle, but animals recovered after the treatment period (Figure 5C). Twenty percent weight loss is considered to be acceptable per the Pediatric Preclinical Testing Program (PPTP) protocol used in this study (10, 17). One animal treated with the combination therapy did not tolerate the treatment and was found dead the week after completing treatment. TMZ at the same dose only resulted in a loss of 4.6% body weight during the treatment period, while NanoTLZ elicited no weight loss throughout the study.

The combination of NanoTLZ and TMZ significantly extended the overall survival compared to the vehicle control and single agent groups (Figure 5D). The median survival time was 11–14 days in the control groups compared to 63 days in the combination group (^**^p < 0.01). At the end of the observation period 4/10 mice treated with NanoTLZ and TMZ had no palpable tumors.

## Discussion

The combination of TLZ and TMZ has demonstrated substantial activity in a number of ES models, however, toxicity necessitated TMZ dose reduction. In order to bypass some of the limitations associated with oral drug delivery a nanoformulation of TLZ, NanoTLZ, was assessed in two different xenograft models. *In vitro* comparison of TLZ and NanoTLZ demonstrated NanoTLZ was as potent if not more potent than TLZ, as evidenced by the IC_50_ values. Both TLZ and NanoTLZ potentiated the effect of TMZ in TC71 cells, though TLZ was more efficient than NanoTLZ. Together, these results indicated NanoTLZ is of similar potency to free TLZ and should be assessed *in vivo. In vivo* imaging demonstrated that NanoTLZ preferentially accumulates in tumors, likely through the EPR effect, and presents a pronounced target inhibition effect. This suggests that more drug may be delivered to the tumor resulting in less drug accumulation in other organs. It was expected this would decrease the systemic toxicity observed with oral TLZ delivery.

KT-10 xenografts have demonstrated dose dependent response to single agent TLZ and therefore, this model was utilized in order to ensure NanoTLZ maintained efficacy *in vivo*. Both NanoTLZ at 1 mg/kg SID and oral TLZ at 0.33 mg/kg BID induced similar responses. However, 3/10 of animals treated with NanoTLZ exhibited a CR with 1/3 MCR until the end of the study, while only 1/10 of animals treated with oral TLZ exhibited a CR. It is important to note that animals receiving NanoTLZ treatment received 33% more drug daily than those on oral TLZ treatment because NanoTLZ was found to be more tolerable than oral TLZ. This higher dose is likely one factor contributing to the enhanced response rate. PARP inhibitors have been shown to exhibit a better anti-tumor effect when PARP is at least 90% inhibited (24, 25). One strategy for achieving long-term inhibition is twice daily administration, as was done with the oral treatment. Previous studies have demonstrated plasma drug concentrations after a single dose of NanoTLZ can be fit with a two compartment model yielding a terminal half-life of 37.5 h (22). The extended half-life of the nanoformulation lead to a similar antitumor effect with only a single injection daily, compared to the twice daily oral administration.

Although ES cell lines were found to be sensitive to PARP inhibitors *in vitro*, the lack of *in vivo* translation necessitated the need to develop rational combinations. The PPTP previously demonstrated 6/10 ES xenografts were sensitive to the combination of TLZ and TMZ (17). Two different combination doses were found to be tolerable when combining the two oral drugs, but both required substantial dose reduction of either TLZ or TMZ. Previous tolerable doses were 30 mg/kg TMZ SID x 5 + 0.1 mg/kg TLZ BID x5, or 12 mg/kg TMZ SID x 5 + 0.25 mg/kg TLZ BID x5 (17). Toxicity testing with NanoTLZ in combination with oral TMZ indicated the combination was better tolerated than the combination of the two free drugs, allowing each drug to be delivered at a higher dose than in the previous study. The combination of 1 mg/kg NanoTLZ administered on days 1, 3, and 5, with 50 mg/kg TMZ SID x5 induced a response in all tumors with a PR in 4/10 and MCR in 4/10 tumors 12 weeks post treatment initiation. Although little weight loss was observed during the MTD testing this treatment regimen did induce acute weight loss (<20%) during the treatment period in the efficacy study, but this was reversed when the treatment ended. The MTD testing was conducted in tumor-free mice and differences in weight loss may be attributed to the shrinking tumors or the presence of the tumors themselves, both of which may affect body weight. The combination of NanoTLZ and oral TMZ significantly extended overall survival compared to each of the single agent controls.

The data presented here demonstrates that changing the delivery system from oral TLZ to NanoTLZ provides an opportunity to modify the dosing required for combination therapy. NanoTLZ was tolerated at a higher total dose compared with free TLZ, and allowed combination with higher doses of TMZ. The KT-10 data demonstrated NanoTLZ administered once daily at a high dose achieved a similar response to twice daily lower dosing, indicating the pharmacokinetics had been altered. The combination of NanoTLZ and TMZ in TC-71 xenografts was promising, but perhaps a better response could be elicited with a lower dose of NanoTLZ administered daily.

NanoTLZ demonstrates similar activity *in vivo* as oral TLZ, but only requires once daily dosing rather than twice daily. It is better tolerated than the oral formulation, which allows for higher doses to be administered. NanoTLZ administered every other day for 5 days effectively potentiated the effect of daily TMZ treatment with 40% of animals being tumor free after 12 weeks. The combination of oral TLZ and TMZ has previously demonstrated both preclinical and clinical toxicity; therefore, NanoTLZ can provide greater versatility in further exploring the best way to limit systemic toxicity while maximizing the effect of this combination.

## Data Availability Statement

The datasets generated for this study are available on request to the corresponding author.

## Ethics Statement

The animal study was reviewed and approved by the Institutional Animal Care and Use Committee (IACUC) reviewed and approved at the University of Texas Health at San Antonio or Northeastern University.

## Author Contributions

PB, RK, and SS designed the research. PB, RC, NB, JC, and RL performed the research. PB and RK analyzed the data. PB wrote the manuscript. RK and SS edited the manuscript.

### Conflict of Interest

The authors declare that the research was conducted in the absence of any commercial or financial relationships that could be construed as a potential conflict of interest.
